# Supplementary figures and images for: Synergy and antagonism in the integration of BCR and CD40 signals that control B-cell population expansion
Source: Mol Syst Biol. 2025 Jun 5;21(8):1119–46. doi: 10.1038/s44320-025-00124-2 (PMC12322056; doi:10.1038/s44320-025-00124-2)

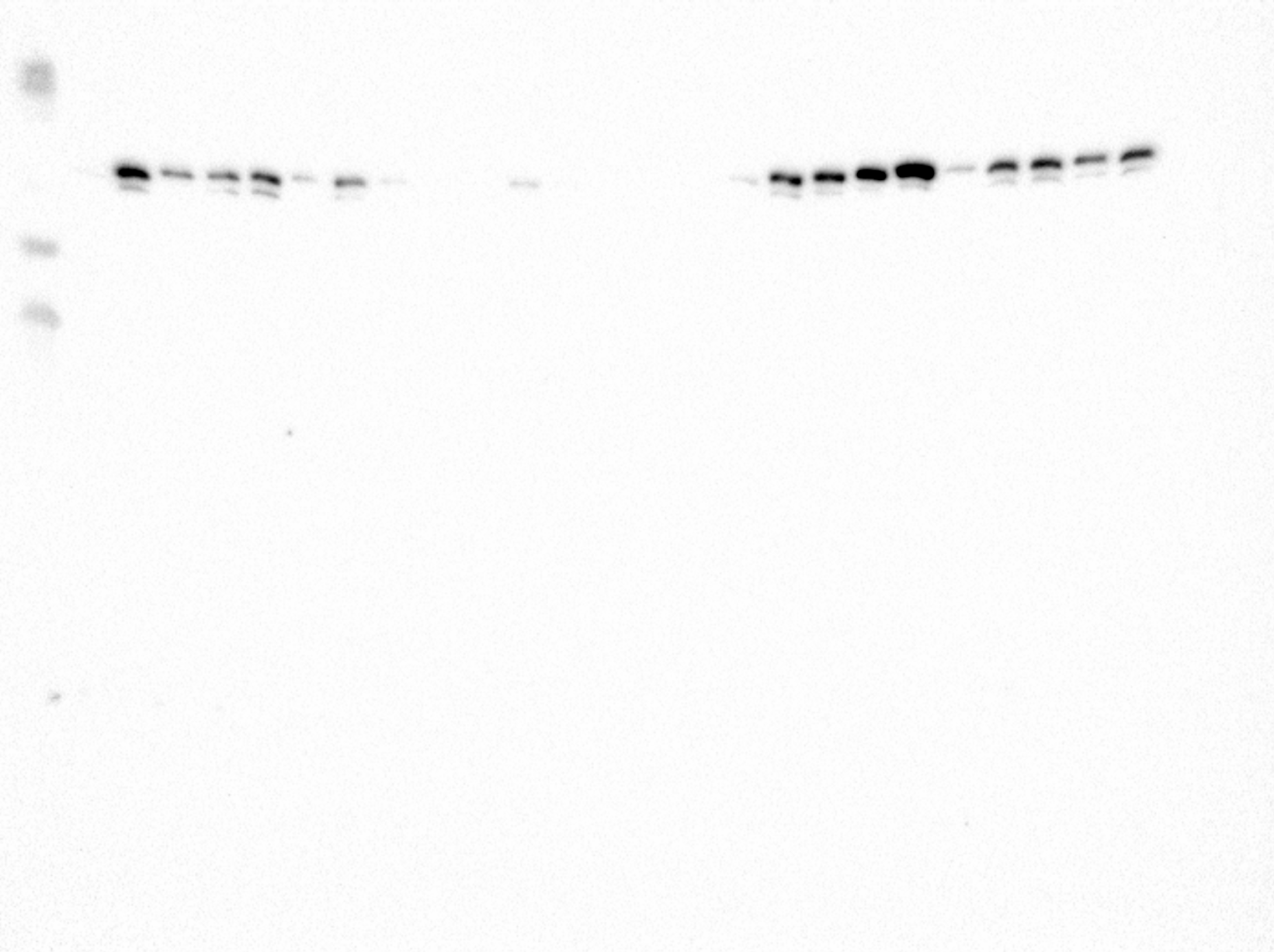

Supplement: Supplementary file 4 — Source data Fig. 1 [file 44320_2025_124_MOESM4_ESM.zip › Figure 1/1H/Western_cRel.tif]

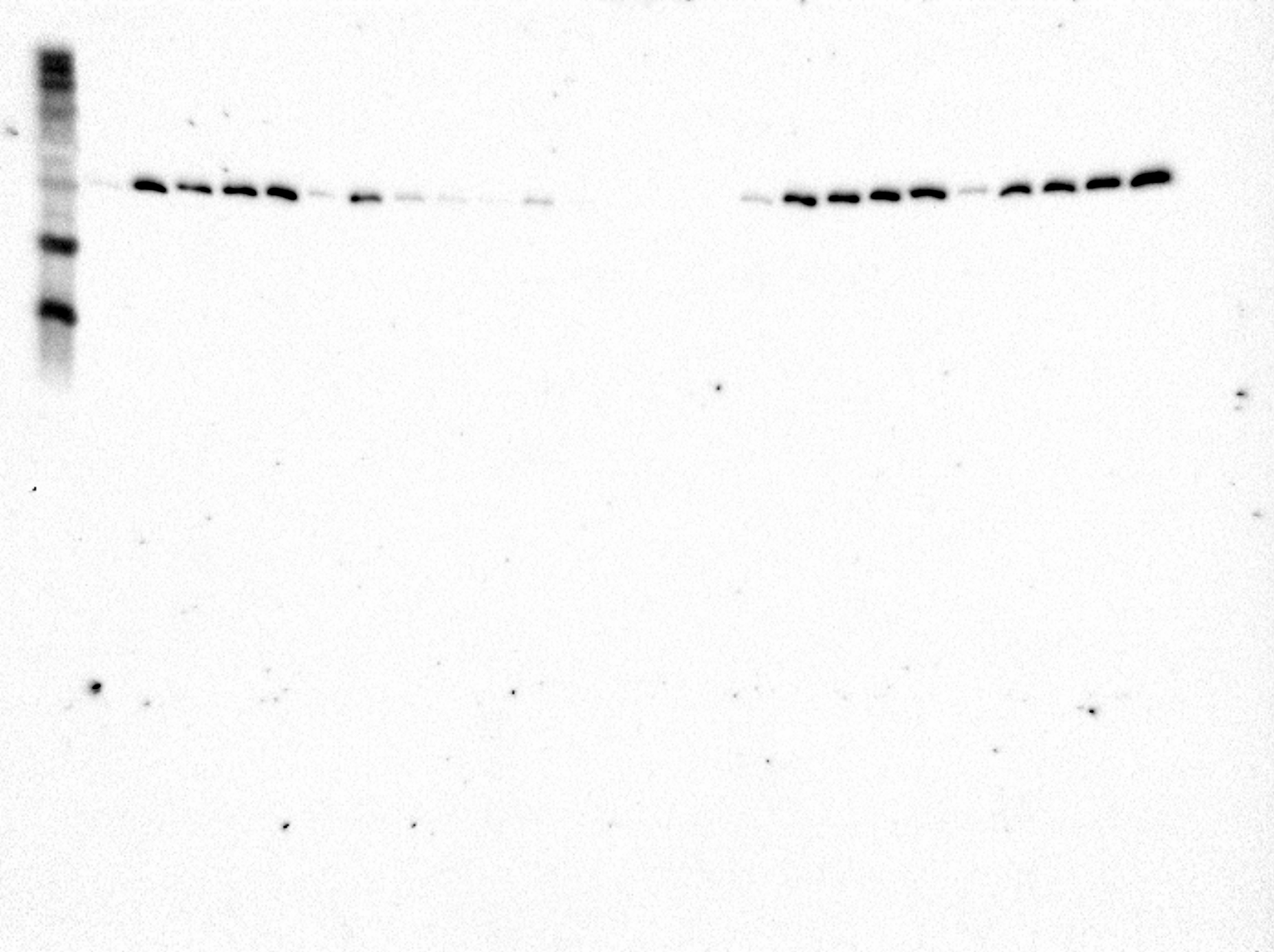

Supplement: Supplementary file 4 — Source data Fig. 1 [file 44320_2025_124_MOESM4_ESM.zip › Figure 1/1F/Western_RelA.tif]
