# Supplementary material for: Synergy and antagonism in the integration of BCR and CD40 signals that control B-cell population expansion
Source: Mol Syst Biol. 2025 Jun 5;21(8):1119–46. doi: 10.1038/s44320-025-00124-2 (PMC12322056; doi:10.1038/s44320-025-00124-2)
Supplement: Supplementary file 10 — Expanded View Figures [file 44320_2025_124_MOESM10_ESM.pdf]

Expanded View Figures

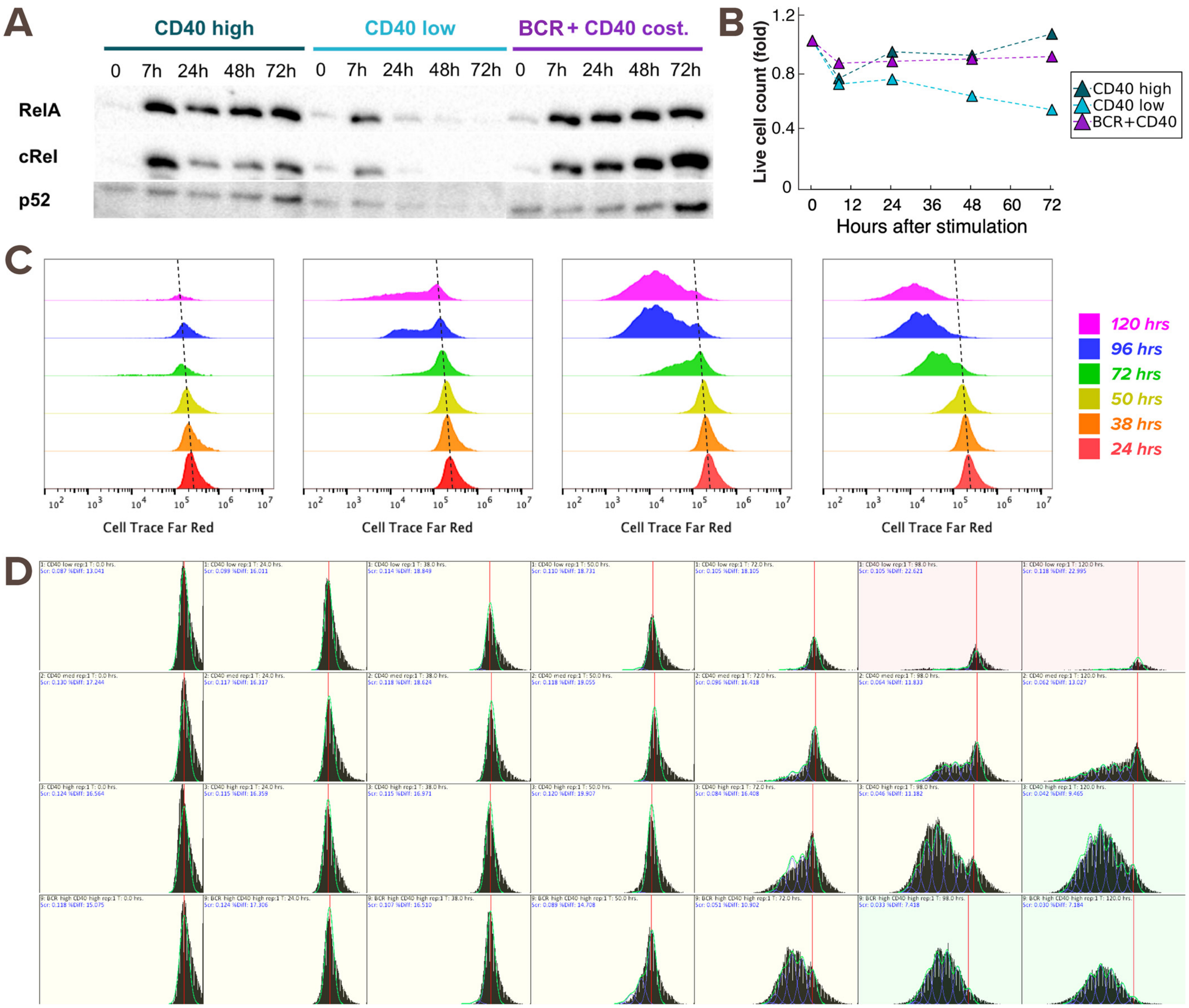

Figure EV1. Raw experimental data to test the multi-scale B-cell model.

(**A**) Immunoblot from experiments with 600 K founder B-cells show nuclear RelA, cRel, and p52 levels at 0, 7, 24, 48, and 72 h after stimulation with low α-CD40 (1 μg/mL), high α-CD40 (10 μg/mL), or costimulation with high α-CD40 and α-BCR (10 μg/mL). (**B**) Line graph of live B-cell count (fold-change) for each timepoint in (**A**), to which the samples are adjusted when loading to the gel. The cell count fluctuation is due to cell death, cell division, and technical error when transferring cells. (**C**) Cell Trace Far Red (CTFR) dye dilution fluorescence histogram for B-cells stimulated with (from left to right) low (1 μg/mL), medium (3.3 μg/mL), and high (10 μg/mL) dose of α-CD40 and costimulation of high α-CD40 and α-BCR (10 μg/mL). There is a baseline shift in CTFR fluorescence by about 2-fold from 24 h to 120 h (dotted line), which we adjusted when deconvolving the cells into each generation. (**D**) Deconvolution of the time courses in (**C**) into each generation, where the red line indicates the center of the undivided population of cells, the blue lines indicate individual proliferation peaks, and the green line represents the model sum.

 © The Author(s)

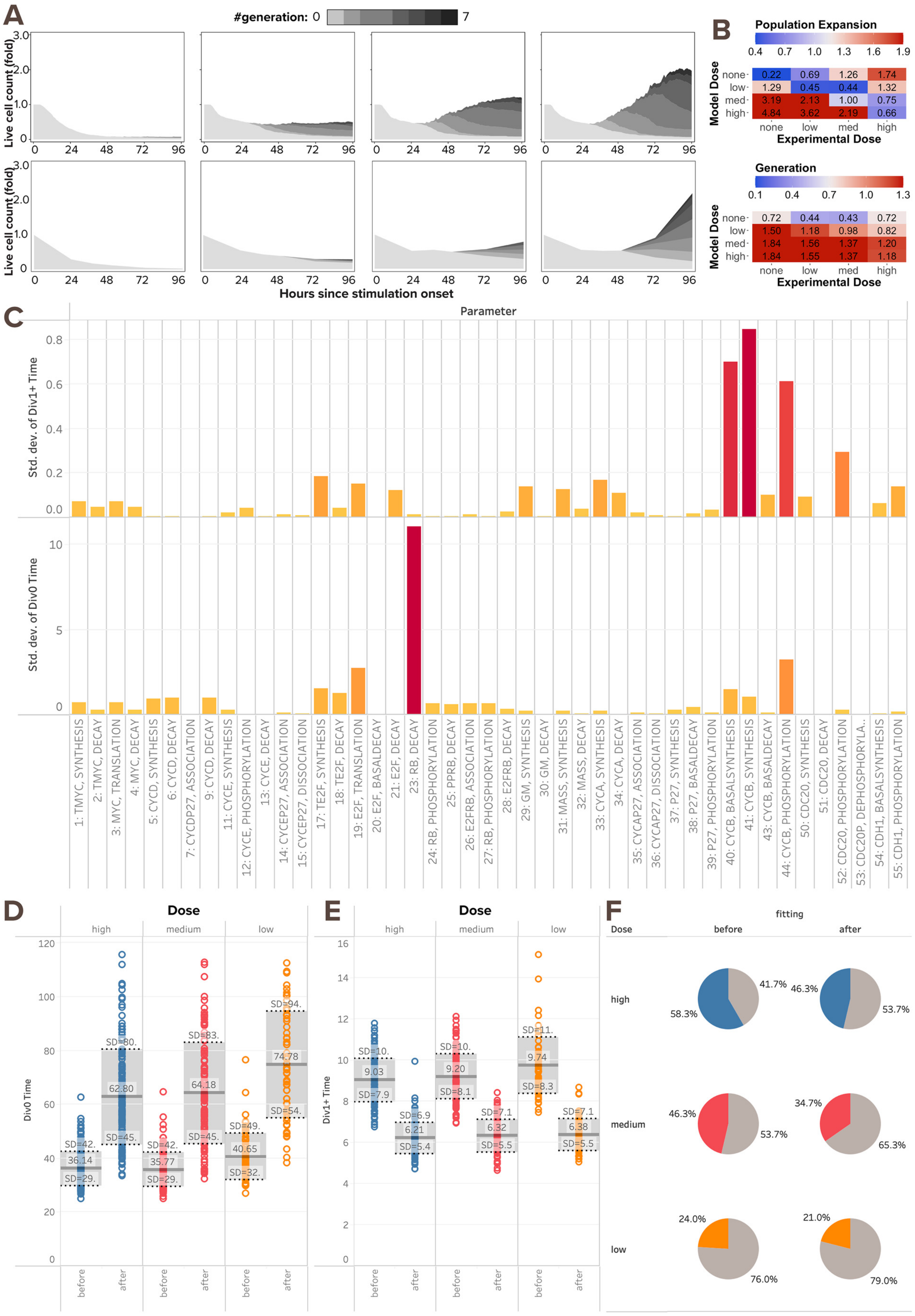
A
#generation: 0 7
Live cell count (fold)
Hours since stimulation onset
B
Population Expansion
0.4 0.7 1.0 1.3 1.6 1.9
none 0.22 0.69 1.26 1.74
low 1.29 0.45 0.44 1.32
med 3.19 2.13 1.00 0.75
high 4.84 3.62 2.19 0.66
Model Dose
none low med high
Experimental Dose
Generation
0.1 0.4 0.7 1.0 1.3
none 0.72 0.44 0.43 0.72
low 1.50 1.18 0.98 0.82
med 1.84 1.56 1.37 1.20
high 1.84 1.55 1.37 1.18
Model Dose
none low med high
Experimental Dose
C
Parameter
Std. dev. of Div1+ Time
Std. dev. of Div0 Time
1: TMYC, SYNTHESIS
2: TMYC, DECAY
3: MYC, TRANSLATION
4: MYC, DECAY
5: CYCD, SYNTHESIS
6: CYCD, DECAY
7: CYCDP27, ASSOCIATION
9: CYCD, DECAY
11: CYCE, SYNTHESIS
12: CYCE, PHOSPHORYLATION
13: CYCE, DECAY
14: CYCEP27, ASSOCIATION
15: CYCEP27, DISSOCIATION
17: TE2F, SYNTHESIS
18: TE2F, DECAY
19: E2F, TRANSLATION
20: E2F, BASALDECAY
21: E2F, DECAY
23: RB, DECAY
24: RB, PHOSPHORYLATION
25: PPRB, DECAY
26: E2FRB, ASSOCIATION
27: RB, PHOSPHORYLATION
28: E2FRB, DECAY
29: GM, SYNTHESIS
30: GM, DECAY
31: MASS, SYNTHESIS
32: MASS, DECAY
33: CYCA, SYNTHESIS
34: CYCA, DECAY
35: CYCAP27, ASSOCIATION
36: CYCAP27, DISSOCIATION
37: P27, SYNTHESIS
38: P27, BASALDECAY
39: P27, PHOSPHORYLATION
40: CYCB, BASALSYNTHESIS
41: CYCB, SYNTHESIS
43: CYCB, BASALDECAY
44: CYCB, PHOSPHORYLATION
50: CDC20, SYNTHESIS
51: CDC20, DECAY
52: CDC20, PHOSPHORYLATION
53: CDC20P, DEPHOSPHORYLA..
54: CDH1, BASALSYNTHESIS
55: CDH1, PHOSPHORYLATION
D
Dose
high medium low
Div0 Time
before after
E
Dose
high medium low
Div1+ Time
before after
F
fitting
Dose before after
high 58.3% 41.7% 46.3% 53.7%
medium 46.3% 53.7% 34.7% 65.3%
low 24.0% 76.0% 21.0% 79.0%

◀ **Figure EV2. Multi-scale model needs tuning to recapitulate B-cell population dynamics in response to CD40 stimulation.**

(**A**) Stacked area plots from model simulations of 1000 virtual B-cells (top) and matching experiments with 19196 founder B-cells (bottom) show their population dynamics in response to stimulation with (from left to right) no (0 nM and 0 μg/mL), low (6 nM and 1 μg/mL), medium (12 nM and 3.3 μg/mL), and high (30 nM and 10 μg/mL) dose of α-CD40. Each subsequent generation of proliferating cells is indicated with a darker gray. (**B**) Heatmap shows RMSD of relative population size expansion (top) and generation composition (bottom) in matching (diagonal) or mismatching (off-diagonal) model-and-experiment pairs. Some model doses (medium and low) are more deviated from their matching than mismatching experimental doses (high and medium, respectively), indicating a subpar fit. (**C**) Bar graph from local sensitivity analysis of parameters in the cell cycle module shows their standard deviations in time to first division (Tdiv0) and time to later divisions (Tdiv1+). Local sensitivity analysis is achieved by repetitive simulations that independently scaling each parameter in the cell cycle module by 0.2, 0.33, 0.4, 0.5, 0.66, 1.0, 1.5, 2.0, 2.5, 3.0, or 5.0-fold. 2 out of 55 parameters stand out as the best candidates for tuning Tdiv0 and Tdiv1+: retinoblastoma (Rb) decay rate and cyclin B (CycB) synthesis rate, respectively. These parameters were tuned in order to achieve a later and more dose-responsive Tdiv0, shorter Tdiv1+, and smaller divider percentage. (**D**) Box plots from model simulations of 300 virtual B-cells show the mean Tdiv0 increases for all doses after parameter tuning. (**E**) Box plots from model simulations of 300 virtual B-cells show the mean Tdiv1+ decreases for all doses after parameter tuning. (**F**) Pie charts from model simulations of 300 virtual B-cells show the percentage of dividing cells (colored slices) out of all founder cells decreases for all doses, while maintaining CD40 dose-responsiveness. Gray slices are the non-dividing founder cells that either die or survive without division.

 © The Author(s)

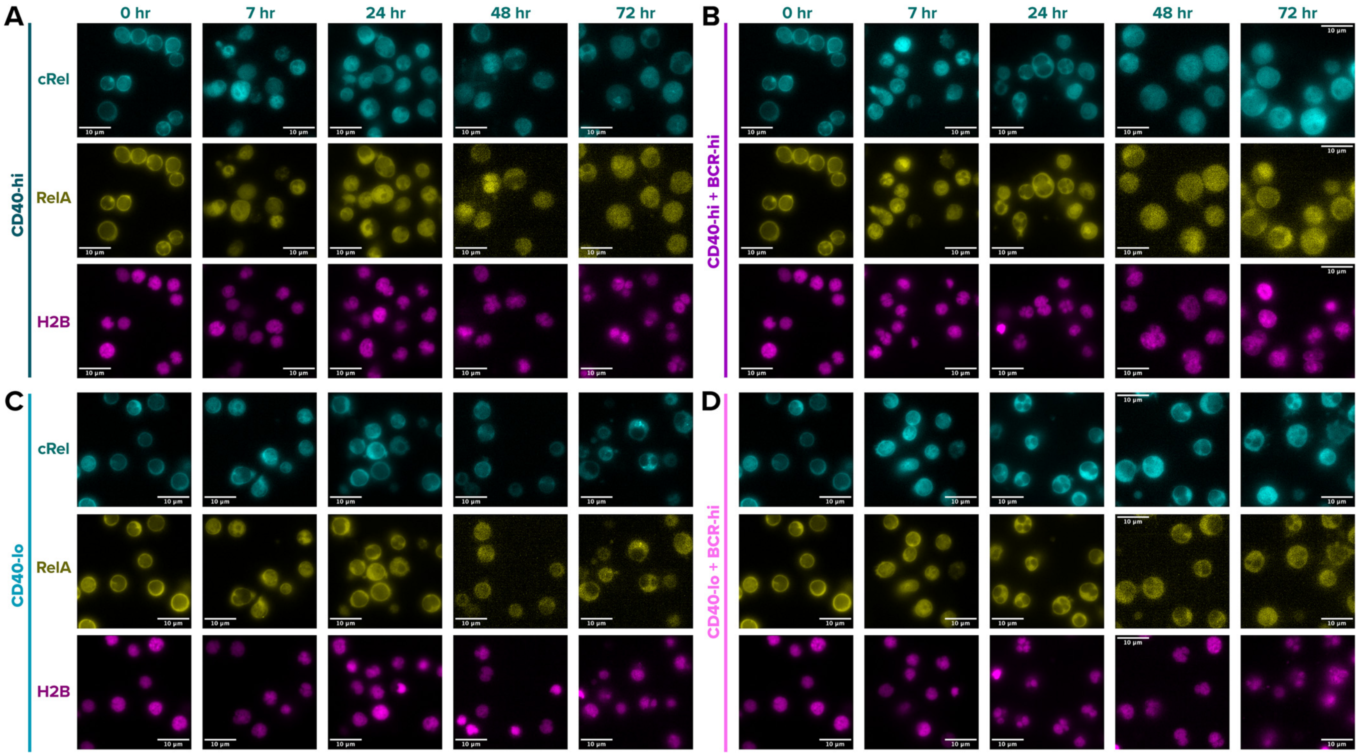

Figure EV3. Batch-normalized, background subtracted multi-channel microscopy captures single-cell NFκB dynamics.

(**A–D**) Multi-channel fluorescence microscopy images of live triple-reporter B-cells at 63X/NA1.4 under oil immersion. Panels from top to bottom show mTFP1-cRel (blue) and mVenus-RelA (yellow) cellular localization, with H2B-mCherry (pink) as a marker distinguishing nuclear from cytoplasmic compartment, all compared from left to right at 0 h baseline, 7 h, 24 h, 48 h, and 72 h post-stimulation with (**A**) high α-CD40 (10 μg/mL), (**B**) high α-BCR (10 μg/mL) and high α-CD40 (10 μg/mL), (**C**) low α-CD40 (1 μg/mL), and (**D**) high α-BCR (10 μg/mL) and low α-CD40 (1 μg/mL).

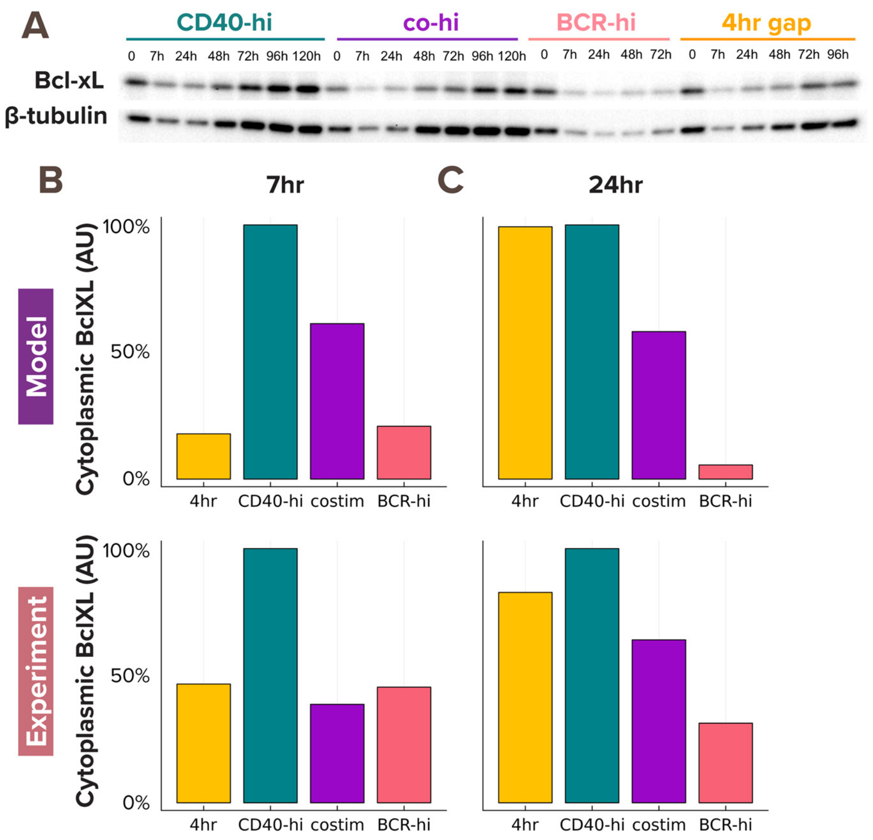

Figure EV4. Model-simulated cytoplasmic BclXL level recapitulates experimental results.

(**A**) Immunoblot from experiments with 600 K founder B-cells show cytoplasmic Bcl-xL and β-tubulin levels in response to stimulation with (from left to right) high (10 μg/mL) dose of α-CD40, high α-CD40 and high α-BCR, high α-BCR, and sequential stimulation of high α-BCR and high α-BCR with a 4 h delay. (**B**, **C**) Bar graphs from model simulations (top) and experiments (bottom) show consistent max-normalized quantification of cytoplasmic Bcl-xL level at (**B**) 7 h and (**C**) 24 h.

 © The Author(s)
